# Positive patient experiences in an Australian integrative oncology centre

**DOI:** 10.1186/1472-6882-14-158

**Published:** 2014-05-14

**Authors:** Bonnie J Furzer, Anna S Petterson, Kemi E Wright, Karen E Wallman, Timothy R Ackland, David JL Joske

**Affiliations:** 1 The University of Western Australia, M408, 35 Stirling Hwy, Crawley, WA 6009, Australia; 2 SolarisCare Foundation Collaborative Research Team, PO Box 7144, Shenton Park, WA 6008, Australia; 3 Sir Charles Gairdner Hospital, Haematology Care Centre, Ground Floor – E Block, Hospital Ave, Nedlands, WA 6009, Australia; 4 Edith Cowan University, 270 Joondalup Drive, Joondalup, WA 6027, Australia

**Keywords:** Cancer, Patient experiences, Complementary therapy, Supportive services, Survivorship, Quality of life

## Abstract

**Background:**

The purpose of this study was to explore the experiences of cancer patients’ utilising complementary and integrative therapies (CIT) within integrative oncology centres across Western Australia.

**Methods:**

Across four locations 135 patients accessed CIT services whilst undergoing outpatient medical treatment for cancer. Of the 135 patients, 66 (61 ± 12 y; female n = 45; male n = 21) agreed to complete a personal accounts questionnaire consisting of open-ended questions designed to explore patients’ perceptions of CIT. All results were transcribed into nVivo (v9) and using thematic analysis, key themes were identified.

**Results:**

Of the 66 participants, 100% indicated they would “recommend complementary therapies to other patients” and 92% stated “CIT would play a significant role in their future lifestyle”. A mean score of 8 ± 1 indicated an improvement in participants’ perception of wellbeing following a CIT session. Three central themes were identified: empowerment, support and relaxation. Fourteen sub-themes were identified, with all themes clustered into a framework of multifaceted views held by cancer patients in relation to wellbeing, role of significant others and control.

**Conclusions:**

Exploration of patients’ experiences reveals uniformly positive results. One of the key merits of the environment created within the centres is patients are able to work through their cancer journey with an increased sense of empowerment, without placing them in opposition to conventional medical treatment. In order to effectively target integrative support services it is crucial to explore the experiences of patients in their own words and use those forms of expression to drive service delivery.

## Background

Cancer is one of the largest contributors to mortality and morbidity in Australia accounting for 19% of the total burden of disease and 12.5% of deaths [1]. From diagnosis and often for many years post-treatment, cancer presents as a significant stressor for patients impacting on emotion, cognition and social interaction, as well as having physical implications. Consequently support services have emerged as an integral aspect of patient management and patient centred care during and after treatment, with interventions ranging from pharmacological to lifestyle modification, and complementary integrated therapies (CIT).

Of relevance, a study of supportive care strategies across six countries, including Australia, reported 35-60% of adults have used some type of non-conventional treatment or therapy since cancer diagnosis [2]. In Australia, CIT use amongst a cancer population has been reported to be between 22-52%, with 75% having tried more than one therapy and remarkably, 40% not discussing this with their physician [3,4]. Interestingly, a study by Miller and colleagues found that despite low patient disclosure rates, 73-94% of patients felt that CIT would assist conventional treatment [4]. Associated with this, Broom and colleagues reported broad scepticism amongst consultants and a desire to limit patient engagement due to the perceived risks of CIT and the potential adverse clinical effects, despite the majority of consultants admitting that they knew little about what interactions could occur [5].

A review of qualitative studies conducted in cancer patients accessing CIT in a variety of supportive environments, identified a series of consistent themes emerging from patient experiences, including; patient empowerment, incorporating control, support, meaning and mastery [4,6-9]; stress and coping [7,10]; wellbeing or quality of life [8,11,12]; connection [8,13,14]; and, integration and polarisation [15,16]. Mok and colleagues found empowerment following cancer support group attendance involved three processes: motivation (meaning in life), mastery over illness (skills and knowledge), and transformation of thoughts (acceptance of illness) [6]. Alternatively, yet inherently linked, Bulsara and colleagues found a ‘fighting spirit’ and support from significant others, along with acceptance, were the key processes in patient coping and empowerment [7].

Importantly, an individual’s style of coping emotionally throughout the cancer continuum can have significant impacts on interpersonal relationships with significant others and medical teams, in addition to treatment tolerance, distress and physiological variables. Perceived preparedness at the end of treatment has been related to greater mood disturbance and health related disease in cancer patients, putting these individuals at an increased risk of psychological distress [17,18]. Conversely, social support and the ability to focus on the positives reduce the occurrence of distress [19].

Following a cancer diagnosis and throughout the entire cancer continuum, patients position themselves in a highly dynamic process along a spectrum from patient to agent. Due to the inherently unequal relationship between non-expert patients and medical specialists it is crucial to develop a patient-centred model of care that incorporates patient autonomy, informed consent and empowerment. Support services that are integrative and complementary to mainstream medical care have been advocated as a means of aiding patient empowerment and autonomy without compromising treatment outcomes. However, in order to be truly integrative supportive services need to be validated within the mainstream medical community and their impact on patients’ psychosocial and physiological outcomes investigated.

Acknowledging the high rates of complementary therapy use among cancer patients and limited institutional integration, it is important to provide an examination of the patients’ experiences within integrative support centres.

Therefore the aim of this study was to explore the personal and unique experiences of cancer patients utilising supportive services. Specifically, this study aimed to examine patients’ perceptions of CIT with respect to novel or emergent themes, along with those previously identified in the literature including wellbeing, support and empowerment. Additionally, we wished to explore what patients’ perceived they gained from accessing services at an integrative oncology clinic.

## Methods

### Participants and setting

The study was conducted over a five-day period at SolarisCare cancer support centres spread throughout Western Australia. The period was selected in conjunction with SolarisCare administrators and researchers and was selected to represent a ‘normal’ week with no special events, therapist absence, school or public holidays. Eligibility requirements for the study included the ability to provide informed consent; a diagnosis of cancer; and presentation to a cancer support centre with the intent to receive services. Of the 135 eligible participants who attended a SolarisCare centre during this 5-day period, 95(70%) completed a questionnaire composed of patient-rated outcomes [20], with 66 of these 95 participants (69%) completing an additional series of open-ended questions designed to explore perceptions of CIT, which forms the focus of this paper.

Participants were provided with a questionnaire to complete independently within one week, which was then returned directly to researchers via a reply paid envelope. This data forms part of a broader study with all individual components granted ethical approval by The University of Western Australia’s Human Research Ethics Committee and the Human Ethics & Research Committee at Sir Charles Gairdner Hospital (RA/4/1/2113/Ref. 2009–61).

As the setting for this study, SolarisCare is a unique cancer support organisation in Australia, offering drop-in centres mostly within hospitals that provide a quiet area, access to information, and a range of CIT for cancer patients and their carers including touch-based, mind-body and energy-based therapies. The centres exhibit a relatively high level of integration with mainstream medical care and the CIT offered are evidence-based or evidence-informed (i.e. CIT guidelines are based on published research in peer-reviewed journals or expert consensus), follow a strict ‘do no harm’ principle and the therapists must have current membership of their National or State organisation. Patients are advised of the services available via their medical team or they can self-refer. All therapies/therapists are constantly evaluated and overseen by a postdoctoral cancer researcher and a haematologist with an extensive background in CIT and patient supportive care. All therapists undergo extensive training to ensure patients’ physical and psychological safety; no major adverse effects have been reported in 12 years of practice. There is no cost incurred by patients for the services.

### Questionnaire

The questionnaire was developed by a multidisciplinary team, which included haematologists, researchers, exercise physiologists, complementary therapists and volunteers. As this study was exploratory in nature, an initial review of qualitative studies was undertaken and anecdotal patient interviews conducted in order to determine and refine the research questions [7,16,21]. A series of open-ended questions were developed, refined and re-worked based on pilot studies with patients of various cancers and treatment stages, and across various ages (Table 1).

**Table 1:** Questionnaire content

**Question**	**Measurement method**	**Previously identified theme**
Q1. How do you feel immediately after a therapy session compared to when you first arrive?	9-point Likert scale	-
Q2. In what ways do you feel better or worse?	Short answer response	-
Q3. How important have complementary therapies been in gaining a sense of control over your own body/health?	Short answer response	Empowerment/sense of control
Q4. Would you recommend complementary therapies to other patients?	Yes or No	-
Q5. If asked by another patient or medical professional what would you say was the best thing you have gained from the complementary therapies?	Short answer response	-
Q6. What has been the biggest challenge?	Short answer response	-
Q7. What impact, if any, do you think complementary therapy has had on your quality of life during or post treatment?	Short answer response	Wellbeing/quality of life
Q8. What other factors, positive or negative do you believe have contributed to your quality of life?	Short answer response	Wellbeing/quality of life
Q9. Will complementary therapies play a significant role in your future lifestyle?	Yes or No	-
Q10. Before you started did you have any concerns/opinions/beliefs regarding complementary therapies?	Short answer response	-
Q11. Do you get support from other individuals who participate in the SolarisCare services or from within the centre? (i.e. emotional, motivational, informational, social)	Short answer response	Support

### Statistical methods

**Figure 1:**
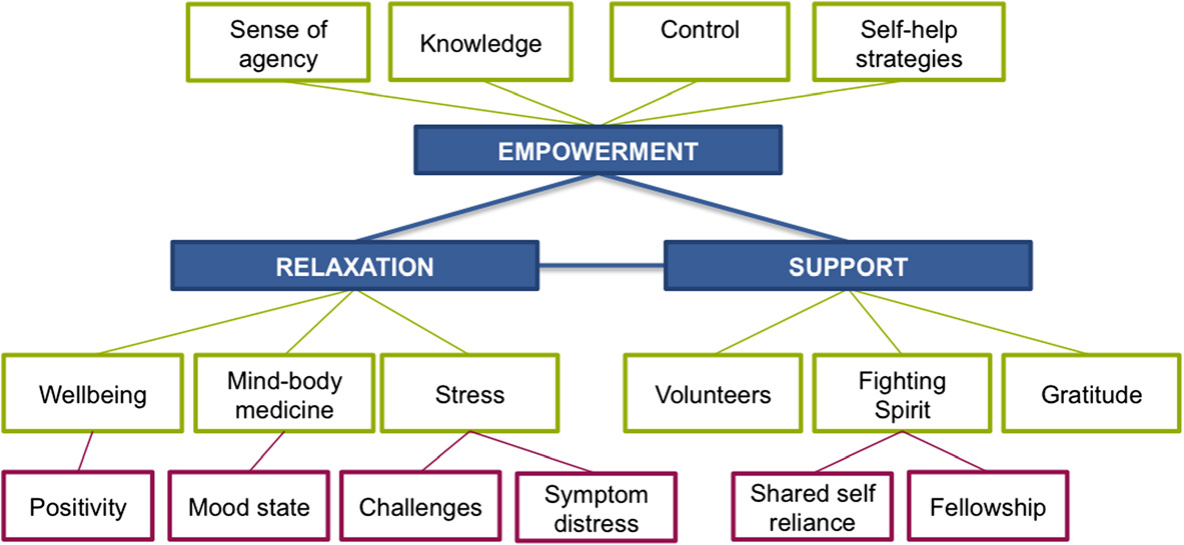
Developed thematic map.

All results were transcribed into nVivo (v9) and subsequently coded into themes using thematic analysis [22]. This allowed for a deductive (themes identified in context of the relevant literature) and an inductive (themes grounded within the data from all participants) analytical approach to be taken via constant comparison. Participants’ responses were coded using a study identification number, gender, age in years, primary diagnosis and question number (i.e. Sn.01, F 50 y, breast, Q1). Key themes were identified via a comprehensive assessment of previous literature and consideration of the research questions, and included empowerment, wellbeing and support. New themes within the data set were identified through data-driven discovery. Line by line and context coding was conducted, and to ensure trustworthiness of the data coding, congruency, concordance and validity, three researchers were involved in the coding process. Once agreement was reached, reclassification and relationships between the sub-themes were determined for grouping into broader central themes and a thematic map developed (Figure 1).

## Results

Of the 66 participants who completed the open-ended questions designed to explore perceptions of CIT, the mean age was 61 ± 12 years (range 39–88 years) with 45 females and 21 males. The participants were diagnosed with a range of cancers including breast (n = 18), colorectal (n = 8), gynaecological (n = 8) and prostate (n = 6), and 42% of participants reported metastatic sites. Additionally, 79% of these participants had at least one other comorbidity.

The most commonly accessed CIT were relaxation massage (56%), reflexology (55%), Reiki (29%) and library/lounge (30%). Additionally, 66% of participants in this trial stated using supplements, with 41% of those self-prescribed. Fifty-three per cent of participants were retired, 17% on sick leave and 18% employed.

Of the 66 participants, 100% indicated they would ‘recommend complementary therapies to other patients’ and 92% stated ‘CIT would play a significant role in their future lifestyle’. Participants rated ‘how they felt immediately after a therapy session compared to when they arrived’ on a scale from one, ‘much worse’, to nine, ‘much better’. A mean score of 8 ± 1 indicated an improvement in participants’ perception of wellbeing immediately following CIT.

Three central themes were identified from participant responses: empowerment (51), support (50) and relaxation (98). Empowerment and support were previously identified deductive themes, supported by our data set and thematic analysis, in addition to the emergent theme of relaxation.

Fourteen sub-themes were identified based on the frequency of participant responses, inductive and deductive analysis (Table 2). These sub-themes were clustered into a framework of multifaceted views held by cancer patients in relation to their wellbeing, the role of significant others, and personal control, within the novel integrative oncology context explored in this study (Figure 1). The themes identified in this study are reported below and supported with representative quotations provided by participants.

**Table 2:** Prevalence of themes in data (n = 66)

**Key concepts**	**First order theme**	**Second order theme**
Relaxation [98]	Stress [24]	Challenges [18]
		Symptom distress [47]
	Mind-body medicine [33]	Mood state [43]
	Wellbeing [44]	Positivity [40]
Empowerment [51]	Sense of agency [18]	
	Control [33]	
	Knowledge [12]	
	Self-help strategies [48]	
Support [50]	Volunteers [57]	
	Fighting spirit [22]	Shared self-reliance [23]
		Fellowship [27]
	Gratitude [40]	

[Total number of participant references to theme].

### Relaxation

There were over 98 independent participant references to the terms ‘relaxation’, ‘relaxed’ or ‘relax’, suggesting that the services within the SolarisCare environment appeared to offer a powerful avenue to elicit a relaxation response:

“Relaxed. If very tired, have more energy and don’t feel as tired. A lot happier mentally. Ready to face the next onslaught of doctors. A good buzz immediately after” [Sn.19, F 56 y, unknown, Q2].

Importantly relaxation is incompatible with feelings of tension, worry and anxiety, all of which are often highly relevant for cancer patients:

“Gained sense of peace, my first session I just cried and the therapy gave me the time to relax and reflect. I know the therapies are doing so much good” [Sn.67, F 67 y, breast, Q5].

Participants also noted the importance of the novel environment that SolarisCare provided within the hospital setting:

“A place to be at peace and relaxed” [Sn.26, M 50y, lung, Q5].

“It is an oasis in the hospital” [Sn.80, F 47 y, gynaecological, Q11].

Contrary to the idea of relaxation is stress or distress, with over 70 responses from participants across the questionnaire referencing ‘stress’ or ‘symptom distress’. However, all described the presence and/or reduction of stress and symptoms following CIT:

“Very very important. Helped me to de-stress, relax and breathe properly” [Sn.68, F 53 y, Brain, Q5].

For many, CIT was perceived to impact them both mentally and physically, in turn aiding their ability to cope with the disease, the side effects of treatment and associated stressors:

“It has been a very big impact both mentally and physically. The therapies are helping my body cope with cancer as well as helping mentally to cope with associated depression. Coping better than I was prior. Mentally I am strengthening all the time” [Sn.19, F 56 y, unknown, Q2].

As noted by one patient following CIT:

“Less tired. Brighter in thinking. Lighter in body. Stronger physically and emotionally” [Sn.33, F 54 y, Breast, Q2].

Many participants noted the impact of CIT on their perceived physical health:

“Less tired. Brighter in thinking. Lighter in body. Stronger physically and emotionally” [Sn.33, F 54 y, Breast, Q2].

Importantly, there were also a number of specific examples of a positive physical outcome following CIT they perceived as ‘relaxing’:

“Calm. Happier. Hopeful for my future. Pain and stress in my body and mind are very much reduced. It lasts for days after therapy. When I started at SolarisCare I was on 8–10 painkillers a day, within four treatments for body pain I was down to 2–4 a day” [Sn.14, F 51 y, Colorectal, Q2].

Notably, participants frequently noted the important link between mind and body:

“It has really helped to make me feel good after feeling so bad. It lifts my spirits therefore lifting my wellbeing and state of mind, as well as relaxing and pampering my body” [Sn.17, F 39, Breast, Q7].

### Empowerment

Complementary integrated therapies are often considered as ‘self-help techniques’ and previous research has associated CIT with increased patient empowerment and an improved sense of control [6,7,10]. As evidenced by the responses below the findings of this study support earlier research:

“When you’re having treatment you follow a prescribed course regardless of how it affects you. Complementary therapies allow you to choose to do something that has positive effects. Feel relaxed, more upbeat, energised. Quality time for yourself and your healing” [Sn.69, F 52 y, Gynaecological, Q3].

Participants frequently described the link between control and other aspects of cancer survivorship:

“My quality of life has improved due to a much-improved sense of control over this stage of my life. When added to greater knowledge and awareness, this generates a sense of empowerment to deal with the challenges ahead” [Sn.41, M 65 y, Prostate, Q7]

In this novel context, participants’ sense of empowerment was enhanced not just due to their increased knowledge and expectations of the disease, but also in their understanding of CIT and the role the therapies play within the cancer experience [16]:

“Therapies have been instrumental in gaining back a sense of control, which I had lost, and was feeling highly anxious about. It is as much that ‘someone cares’, I get that sense from the volunteers, as it is from the therapy itself. Hard to define, but it’s the total service which produces benefits” [Sn.41, M 65 y, Prostate, Q3].

Importantly, rather than opposing traditional medical systems participants perceived the centres’ as working with medical teams:

“I feel I am doing my part in return to full health. I have put my trust in the medical profession to do their bit. Choosing to complement their treatments with therapies I am working with them and taking some responsibility for my wellbeing” [Sn.75, F 65 y, Colorectal, Q5].

With the majority of participants on treatment, these views provide evidence of a unique integrative relationship between the centre, medical consultants and patients who are provided with the ability to take some control and agency during the course of treatment through CIT usage, without alienating their medical teams:

“My doctors are very open to me trying therapies if they give me a sense of wellbeing” [Sn.32, M 64 y, Prostate, Q11].

Importantly, participants perceived their choice to participate in CIT as an active process rather than a passive treatment:

“Feel an active participant in my wellbeing. I feel I’m treated as an individual and there is way more to me than my cancer” [Sn.80, F 47 y, gynaecological, Q5].

### Support

In respect to this, SolarisCare centres provide a multi-tiered approach to patient support. This is demonstrated by the therapists who share their skills and are trained in ‘active listening’ and secondly by the presence of other patients (peers). Additionally, a unique and integral aspect of the centres is that skilled volunteers provide all the services:

“Seeing and attending SolarisCare makes me feel I’m among like minds. People who also live and thrive since their cancer. Inspiring” [Sn.80, F 47, gynaecological, Q11].

The provision of a patient resource and drop-in centre staffed by knowledgeable, like-minded individuals who volunteer their time creates a unique environment of shared experience, as described by participants:

*“Not being alone. Unconditional support and understanding. Knowing I can do something to relieve my systems and address my concerns”* [*Sn.28, F 60 y, breast, Q5].*

Empathy and identification have been shown to be key aspects of a supportive relationship and the most valuable information for patients is that which parallels their cancer experiences [18]:

“All volunteers caring, spent time talking, explaining and sharing their story related to my problem. Brought us a cup of tea and ‘home-like’ comfort. Made journey much more do-able. Encouragement and understanding. Refreshing and great. Personal touch not rushed” [Sn.55, F 72 y, Colorectal, Q11].

In this novel setting, participants described a sense of community, fellowship and care, as one participant explained:

“The moment you walk through the front door of SolarisCare you are made to feel loved, special, cared for and worthy of their (volunteers) time. A wonderful experience at a not some wonderful time of my life. Thank so much for the wonderful facility” [Sn.17, F 39, Breast, Q7].

Contrary to other studies, in this cohort ‘fighting spirit’ was a sub-theme of support rather than empowerment due to the context of patient responses [7]:

“Knowing there are no many people just like you. The volunteers are amazing people. Make you feel good, relaxed and hopeful that you can beat your cancer” [Sn.10, F 67 y, Breast, Q5].

Participants noted the importance of the support they received from those with similar experiences;

“Good to have a place to open up should you want to, without putting extra burden on family and friends. Nice to meet people who are going through what you are. More empathy than in a chemo ward or doctors surgery” [Sn.69, F 52 y, Gynaecological, Q8].

## Discussion

The key thematic findings of this study highlighted CIT, as practiced in the SolarisCare centres, as an opportunity for patients to feel relaxed, supported and empowered, and to take an active role in their health, often for the first time since the diagnosis of cancer [23,24].

Importantly, a number of key themes identified impact patients both physically and mentally. Specifically, a relaxation response refers to the physiological and psychological response that is in direct contrast to the stress response [25]. Physiologically, a relaxation response is defined as “a set of changes in bodily functions such as slowing of the heart rate and breathing rate, and an increase in the brain waves associated with relaxation, that take place as a result of meditating or practicing other relaxation techniques” [25]. Of relevance, researchers have suggested four basic elements are required to elicit a relaxation response, all of which can be facilitated through selected CIT in a purposefully designed environment, as exhibited at selected integrative oncology centres [26].

Based on the responses of participants, CIT at SolarisCare centres provides a counter-balance to the stress elicited by a cancer diagnosis and medical treatment. In-turn, CIT may play an important role in aiding patients to comply with, and tolerate treatment, in order to achieve optimal outcomes [27]. Additionally, short-term improvements in distress can be seen immediately following periods of reduced stress with CIT improving patients’ abilities to cope and regulate emotion [28].

Furthermore, researchers have demonstrated that a stress response is linked to acute and long term neurophysiological changes resulting in further symptoms of distress, as well as negatively affecting cognition and coping skills [28,29]. In turn, active coping strategies are associated with lower cortisol levels and improved wellbeing [28]. The impact of psychological distress and the ability to cope with physical symptoms is a crucial aspect in the consideration of the effectiveness of CIT.

Stress and/or relaxation is also closely linked to mind-body medicine, which is predicated on psychological factors directly influencing physiologic function and health outcomes [30]. Therefore, researchers have proposed that improvements in ‘state of mind’ and psychological coping described by participants following selected therapies may lead to improvements in physical symptoms of distress and treatment tolerance [27,30]. Waldrop and colleagues demonstrated psychological factors such as coping style, emotional states, hostility and stress have been shown to directly influence physiological function and health outcomes [10]. Complementary integrated therapies are often considered as ‘self-help techniques’ and previous research has associated CIT with increased patient empowerment and an improved sense of control [6,7,10]. In turn, the development of a sense of control is associated with improved health and longevity [10].

Being prepared through knowledge, given the coping tools to face stressors, and providing a safe environment to deal with the emotions that come with a cancer diagnosis are regarded as highly valued aspects of integrative centres that allow patients to emerge from sessions feeling prepared, stronger, and in control [18,31]. There is an increasingly high uptake of CIT demonstrated in the literature [2,3]; however consultants’ approaches in discussing CIT with their patients are based around personal beliefs and individual preferences for conflictual versus empowering dynamics in the patient-doctor relationship [5]. As demonstrated in previous research, increases in patient knowledge and control can be seen to create greater equality between patient and consultant, facilitating patient empowerment and a more effective interpersonal dynamic and self-efficacy with regards to medical interventions [18,31,32].

Strong emotions can be modulated through sharing fears and problems with similar others, a mutual understanding that is unique to those who have experienced or worked extensively within cancer [23]. Additionally, fellowship is established through shared self-reliance with volunteers and other patients, in addition to the experience of feeling cared for by others. In the context of the SolarisCare centres the therapists and volunteers are an integral aspect of the experience with patients developing a shared self-reliance and fellowship with other patients and staff. This in turn leads to increased feelings of hope and the ability to have, or maintain, a fighting spirit.

In the community and reinforced by the media, cancer is portrayed as an enemy and invader with those diagnosed obliged to fight and maintain a positive attitude, making them morally culpable for their recovery [23]. The SolarisCare centres provided a safe environment that permitted patients to express emotions without burden.

Additionally, researchers have identified a series of unmet needs amongst cancer patients including desire for information, respite or time away from their carer, help in dealing with emotional problems, ventilation of feelings and dealing with fear of recurrence [33]. The SolarisCare centres are uniquely positioned to address these needs through the provision of a resource library, therapies that allow a ‘time-out’ without fears for safety or health, and support provided by therapists and volunteers:

### Limitations

This study involved the use of open-ended questions as a preliminary exploration of the experiences of patients attending novel integrative oncology centres. Generalisations regarding the findings of this study across other centres offering CIT or individual therapies should be avoided. However, the overwhelmingly positive experience of patients may guide future research and the development of integrative oncology centres aiming to enhance patient support and service delivery. In-turn, this may indirectly drive improvements in clinical outcomes.

The exploration of a patients’ personal experience allows researchers a unique view of integrative oncology centres, however there are acknowledged limitations from utilising a questionnaire-based design. Future research should include in-depth interviewing and focus groups, with the potential for purposeful sampling in order to thoroughly explore given concepts and themes. Additionally, an examination of the experiences of volunteers, therapists and medical professionals with regards to integrative oncology centres would provide invaluable information regarding the highly dynamic relationship involved in integrative, patient-centred care.

## Conclusions

This study demonstrated that the current delivery of supportive care and complementary therapies, enabled patients to work through their personal cancer journey with an increased, sense of empowerment, integrating selected therapies to improve their quality of life, without placing them in opposition to conventional medicine. Also, that psychosocial support from the SolarisCare centres facilitated positive relationships with family, friends and significant others through relieving their burden of care, allowing patients to express emotions in a safe and guilt free environment [32]. Patient reported outcomes from this study will affect the efficacy and enhancement of existing services and in the development of new centres.

## Competing interests

The authors have no financial relationship or conflicts of interest to declare. Authors have full control of all material associated with this paper, and will allow the journal to review data if necessary.

## Authors’ contributions

All authors participated in the conception and design of the study, and have been involved in the drafting and revising of the manuscript for critically important intellectual content. All authors have read and approved the final manuscript. BF, KW and AP coordinated the collection of data and analysis, with BF and AP conducting the analysis and interpretation under the supervision of the entire research team.

## Pre-publication history

The pre-publication history for this paper can be accessed here:

http://www.biomedcentral.com/1472-6882/14/158/prepub
